# Obesity in Polish Children and Parents’ Perception of Their Children’s Weight Status: The Results of the SOPKARD-Junior Study

**DOI:** 10.3390/ijerph19084433

**Published:** 2022-04-07

**Authors:** Klaudia Suligowska, Jacek Buczny

**Affiliations:** 1 Department of Dental Techniques and Masticatory System Dysfunctions, Medical University of Gdańsk, Tuwima Street 15 Office 17A, 80-210 Gdansk, Poland; 2 Department of Preventive Medicine and Education, Medical University of Gdańsk, Dębinki Street 7, 80-952 Gdansk, Poland; 3 Department of Experimental and Applied Psychology, Vrije Universiteit Amsterdam, De Boelelaan 1105, 1081 HV Amsterdam, The Netherlands; j.buczny@vu.nl; 4 Institute of Psychology, SWPS University of Social Sciences at Sopot, Polna 16/20, 81-745 Sopot, Poland

**Keywords:** childhood obesity, parental perception, weight perceptions, BMI, Poland, children, pediatric obesity, early detection, overweight

## Abstract

One way to counteract the spread of obesity in children is its early recognition by parents. Therefore, the aim of this study was to investigate whether parents’ perception of their children’s BMI category was consistent with their actual BMI assessment and to determine potential influential factors. The study was conducted as part of the “A program for the early detection of risk factors for lifestyle diseases SOPKARD-Junior” (SOPKARD-Junior), a preventive health program implemented in public elementary schools from 2017 to 2019. The results from 381 children with a mean age of 11.67 (*SD* = 1.52) were analyzed. Height and weight were measured and BMI was calculated. Surveys were conducted to assess parents’ perceptions of their child’s weight, along with their sociodemographic status. Nearly one in three parents were unable to correctly identify their children’s weight; 25.0% underestimated it, and 6.0% overestimated it. Underestimation was observed along with an increase in the children’s BMI weight category, with as many as 57.1% of parents of obese children misclassifying their weight. The child’s BMI was the only significant predictor of incorrect assessment. Polish parents cannot properly assess their children’s BMI, especially when their child is overweight or obese. In children, weight and height measurements should be taken regularly, rather than allowing weight category to be dependent upon parental weight assessments.

## 1. Introduction

Childhood obesity is a serious disease, as it is one of the risk factors for non-communicable diseases (NCDs), e.g., cardiovascular, endocrine, musculoskeletal, pulmonary, and psychological diseases, that often intensifies with increasing obesity [1,2,3]. In the World Health Organization (WHO) European Region, one in three children is overweight. It is predicted that more than 60.0% of children who are overweight before puberty will be overweight in early adulthood [4]. The prevalence of this disease is growing rapidly all over the world, and despite many efforts, no effective method has been found to stop this epidemic [5].

It seems that one of the first steps to counteract the spread of abnormal body weight is the early identification of the existing problem. In children and adolescents, a key role in this process is played by parents, whose involvement can influence a change in their children’s lifestyle [6]. Parents who misclassify their children’s body mass index (BMI) may have less knowledge and parental self-efficacy in regard to healthy behaviors [7]. The appropriate and timely recognition of when a child’s BMI or weight category falls just outside the age norm would allow for early educational, preventive, and therapeutic interventions. Such a strategy would be effective in preventing the development of the severe consequences of obesity.

However, many studies indicate that parents are unable to correctly assess their children’s weight and weight category. A meta-analysis revealed that the underestimation of obesity in children ranged from 13.3% to 100% [8]. Studies related to the factors affecting the perception of a child’s weight are inconclusive. Some studies have shown that a child’s gender, age, BMI, and socioeconomic status, and their mother’s and father’s education levels, can help to predict parental perception, whereas others have not shown such implications [8,9]. Examining parents’ perceptions of their children’s BMI category may help in understanding the ever-increasing spread of obesity. As the present relationships are understudied in Poland, we expect that the current study will also contribute to the research conducted in other countries.

BMI (calculated from body weight and height) has become an accepted screening tool for assessing body weight in children and adults. The BMI is the basis of the body weight classification system and surveillance statistics. This test is inexpensive and simple, but it also has its limitations [10,11,12,13]. This test does not account for physiological variations in the proportion of fat and muscle mass and the distribution of body fat [14,15]. Therefore, it may not be a reliable diagnostic tool in the case of individuals for whom attention should be paid as to the size and distribution of body fat, e.g., athletic, sarcopenic, TOFI (Thin Outside Fat Inside) and FOTI (Fat Outside Thin Inside) phenotypes. This means that some lean individuals with preserved muscle mass (which does not suggest poor health) may be labeled as overweight. Additionally, patients with a normal BMI who have an abnormal distribution of body fat, and thus are at increased risk for metabolic disease, would not be considered at risk according to BMI criteria [10,13,16,17,18,19].

The main objective of this study was to explore whether parents’ perception of their children’s BMI category is consistent with the actual assessment of BMI. In addition, we tested whether the children’s gender, age, BMI, the existence of siblings, parental education, and family affluence were correlated with the accuracy of the parents’ perception. Our goal was to provide additional evidence to facilitate the global understanding and prevention of obesity in children.

## 2. Materials and Methods

### 2.1. Sample and Procedure

The research was conducted within the preventive program “A program for the early detection of risk factors for lifestyle diseases SOPKARD-Junior” (SOPKARD-Junior), the main purpose of which was a comprehensive assessment of the health status of children and adolescents [20,21,22]. The program was implemented in 2017–2019 from September to December each year in elementary schools within the city of Sopot in the Pomeranian Province of Poland (approximately 35.5 thousand residents). In 2017 and 2018, all 5th grade students at Sopot elementary schools were invited to join the program, and in 2019, all 8th grade students were invited to join. The study began after receiving written informed consent from each child’s parent/legal guardian, as well as approval from the bioethics committee of the Medical University of Gdańsk. The organization and supervision of the methodology of all the studies were carried out by one experienced person with a specialization in public health (project manager and principal investigator).

There were 582 children invited to the study, 527 of which received consent to participate. However, not every parent delivered the requested data, and thus, due to the lack of information for variables used in this study, 146 participants were removed from the analyses. Importantly, the loss in data was random. The final sample size of this study was 381. The mean age of the children was 11.67 (*SD* = 1.52; range from 10 to 16 years); 55.4% were girls and 44.6% were boys. Of these children, 5.2% were underweight, 68.5% were normal weight, 18.6% were overweight and 7.6% were obese. Higher education was present in 61.9% of mothers and 53.0% of fathers. A medium level of family affluence was found in almost half of the participants. A statistically significant difference between boys and girls was found only in BMI, where obesity was found in every 10th boy (B) and every 28th girl (G), whereas being underweight was found less frequently in boys (B: 2.8% vs. G: 8.2%).

### 2.2. Anthropometric Measurement

Anthropometric measurements, including height and weight, were taken on school premises by trained researchers. Body height was measured standing in an upright position, without shoes, with an accuracy of 1 mm, using a portable stadiometer (Leicester Height Measure). BMI was measured with the use of the RADWAG WPT 100/200 O scale with an accuracy of 100 g. BMI was calculated based on the formula dividing weight (kilograms) by height (meters) to the square. Underweight, normal weight, overweight, and obesity were classified using WHO-recommended sex- and age-specific cut-off *z*-scores for BMI [23]. Specifically, we used charts and tables with the *z*-scores to determine the BMI category for every boy and girl in the study, following the 2007 WHO-recommended growth references for children and adolescents [24]. If a child’s BMI score was equal to a *z*-score of −2 or lower, then BMI was categorized as underweight; if the BMI value was equal to a *z*-score between −1.99 and 1, then BMI was categorized as normal weight; if the BMI value was equal to a *z*-score between 1.01 and 2, then BMI was categorized as overweight; and lastly, if a BMI score was equal to a *z*-score of 2.01 or higher, then BMI was categorized as obese.

### 2.3. Questionnaire Distribution and Demographics

The questionnaires were delivered to all parents whose children participated in the SOPKARD-Junior program. The parents had 3 weeks to complete the questionnaires and submit them to the study’s principal investigator. All the responses were encoded to ensure anonymity.

Having a sibling was determined by the question: “Does the child have a sibling?”; “Yes” was coded as “1”and “No” as “0”.

To determine the parents’ education level, two questions were used: “Education of the child’s mother/legal guardian (please, underline correct option)” and “Education of the child’s father/legal guardian (please, underline correct option)”. The possible answers were: (1) incomplete primary, (2) primary, (3) lower secondary, (4) basic vocational, (5) incomplete secondary, (6) secondary, (7) post-secondary, (8) incomplete higher education, and (9) higher education. To simplify the analysis, education levels (1)–(3) were classified as “lower than high school”, (4)–(8) were classified as “high school”, and (9) was classified as “university level”.

### 2.4. Family Affluence Scale

The Family Affluence Scale (FAS) derived from the Health Behavior in School-Aged Children (HBSC) questionnaire recommended by the WHO was used in order to assess family wealth. The questionnaire was completed by the participants [25]. HBSC is an international, worldwide survey with 47 countries as its members. It is a validated tool recommended for determining family affluence [26,27]. The FAS used in this study consisted of six items (version III [26,27]); based on the questionnaire results and following the categorization rules [26,27], we identified three levels: (1) “lower family affluence” for scores 0–6, (2) “medium family affluence” for scores 7–9, and (3) “higher family affluence” for scores 10–13. Table 1 shows that the majority of the participants were in the medium family wealth group, whereas the lower family wealth group included the smallest number of participants.

### 2.5. Parental Perception of Children’s Weight Category

Parental perception was assessed with the question: “How would you rate your child’s weight?” There were five options: “significantly underweight”, “slightly underweight”, “normal weight”, “slightly overweight”, and “significantly overweight”. For consistency in terms of the presentation of the results, the responses were coded into the following categories: (1) underweight (“significantly underweight”, “slightly underweight”), (2) about right/normal weight (“normal weight”), (3) overweight (“slightly overweight”), and (4) obese (“significantly overweight”).

### 2.6. Statistical Analyses

A descriptive analysis (percentages, means, and standard deviations) and statistical inference techniques were carried out in order to describe the relationship between the tested variables.

For the purpose of the main statistical analyses, three variables were created: (1) Parental misconception of children’s weight category: if the parental perception reflected the correct WHO category, the result was coded as “0” and the value of “1” was coded if otherwise. Only “1” was counted to represent the level of misconception. (2) Accuracy of parental perception of children’s body weight: if a parent underestimated the weight category in comparison to the WHO category, the score of “−1” was coded; if accurate, then “0”, but if overestimated, then “1” was coded. (3) Parents’ accuracy in identifying children’s weight classification: if accurate, then the result was coded as “1”; if inaccurate, then “0” was coded.

To analyze contingency tables (frequencies per category), χ^2^ and Cramer’s *V* were used to reflect the effect size. Values of *V* indicated the effect size as follows: between 0.00 and 0.10 = very low, 0.11–0.30 = low, 0.31–0.50 = medium, and 0.51–1.00 = high. In addition, κ (kappa) was calculated to reflect the level of agreement between the objective BMI categories and parental perception. The values reflected the following: between 0.00 and 0.20 = poor agreement, 0.21–0.40 = fair agreement, 0.41–0.60 = moderate agreement, 0.61–0.80 = substantial agreement, and 0.81–1.00 = strong/perfect agreement. A negative κ indicated disagreement.

To compare means, the *t*-test for independent samples was used. The logistic regression model was used to test the predictors of parent’s accuracy in identifying children’s weight classification. The predictors were children’s age, children’s WHO BMI category, the existence of siblings, mother’s and father’s education levels, and family affluence level (FAS). Age was treated as a continuous predictor. All other predictors were entered as categorical predictors with a predetermined reference category.

We based our statistical inference on *p*-values, assuming 0.05 to be the threshold. However, if possible, we reported confidence intervals for the effect sizes to make the analyses complete. All the analyses were performed using SPSS 26 (SPSS and IBM Company, Chicago, IL, USA).

## 3. Results

**Figure 1:**
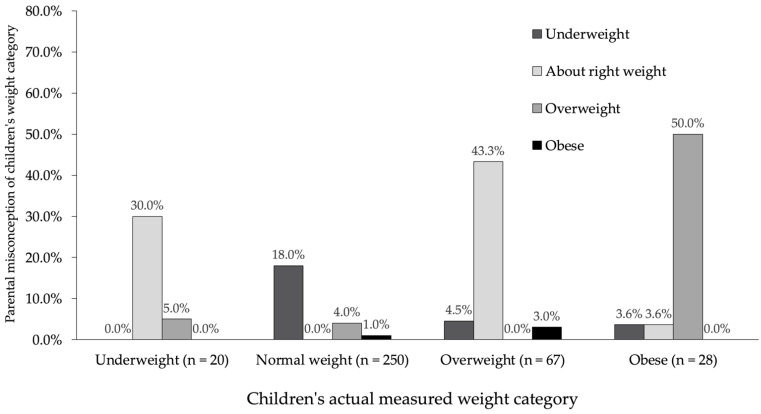
Parents’ misclassification rate by measured weight categories. For instance, 18% of normal-weight children were perceived as underweight.

Figure 1 shows that, within the group of children in the underweight BMI category, 30.0% were misclassified by their parent as having normal weight, while 5.0% were considered overweight. In the group with a normal BMI, the pattern of results was different, revealing that 18.0% of children were misclassified as underweight, and only 4.0% as overweight. However, in the group of participants whose BMI was categorized as overweight, the pattern of misperception was different than in the second group, but similar to the pattern in the first group, where 43.3% of children in the overweight group were considered as normal weight. Finally, in the group categorized as obese, 50.0% of children were miscategorized by their parent as overweight, but no one indicated obesity.

In general, the relationship between the children’s actual measured weight category and the parents’ perception was statistically significant; therefore, there were significant differences between the percentages in the groups (see Figure 1), χ^2^(12) = 266.93, *p* < 0.001, Cramer’s *V* = 0.49, 95% CI for Cramer’s *V* [0.44, 0.58]. There was little disagreement between the measured BMI and parent’s perception, κ = −0.07, *p* < 0.001, 95% CI for κ [−0.09, −0.05]. To summarize, the pattern of results depicted in Figure 1 suggests that parents systematically perceived their children’s weight as lower than the objective measure indicated, but only for normal or higher than normal BMI.

In order to better understand parental perception, we used the factor of children’s sex as a moderator to test how accurately their weight category was estimated by their parents.

**Figure 2:**
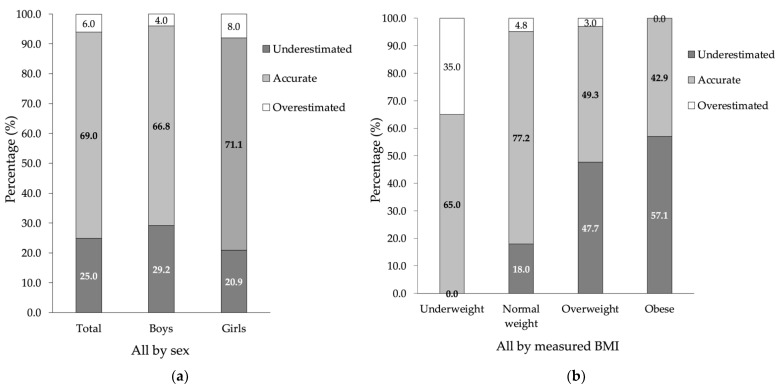
Comparison between measured BMI and parental perception of children’s body weight. (**a**) Accuracy of parental perception of children’s body weight by sex; (**b**) Accuracy of parental perception of children’s body weight by measured BMI.

In total, 31.0% of parents misclassified their children’s weight category, and as many as 25.0% of them underestimated it; however, this was observed more often in boys (29.2%) than in girls (20.9%). Overestimation of children’s weight was observed more often in girls (in 8%) than in boys (4%) (Figure 2a). The relationship between children’s sex and the accuracy of weight estimation was statistically non-significant, χ^2^(2) = 5.24, *p* = 0.073, Cramer’s *V* = 0.12, 95% CI for Cramer’s *V* [0.04, 0.22]; the correlation was very weak. Details can be seen in Figure 2a.

Underestimation was observed with an increase in children’s BMI weight category, with as many as 57.1% of the parents of obese children misclassifying their weight (see Figure 2b). The relationship between measured BMI categories and the accuracy of parental weight estimation was statistically significant, χ^2^(6) = 76.36, *p* < 0.001, Cramer’s *V* = 0.32, 95% CI for Cramer’s *V* [0.24, 0.43], κ = −0.01, *p* = 0.307, 95% CI for κ [−0.03, 0.01]; however, there was no agreement between parental perception in terms of underestimation/accuracy/overestimation and measured BMI. For details, see Figure 2b.

**Figure 3:**
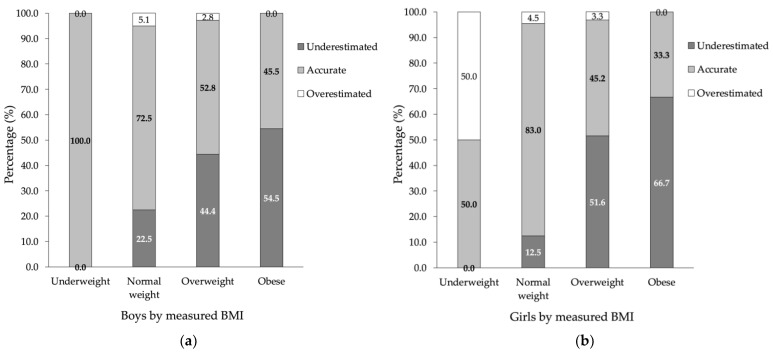
Comparison between measured BMI and parental perception of children’s body weight. (**a**) Accuracy of parental perception of children’s body weight by measured BMI in boys; (**b**) Accuracy of parental perception of children’s body weight by measured BMI in girls.

The increase in children’s weight category estimation was lower in boys (54.5%) than in girls (66.7%); see Figure 3a,b for details. The parents of underweight boys always assessed their children’s weight correctly, whereas as many as 50% of the parents of girls overestimated it. The relationship between the measured BMI category and the accuracy of weight estimation in boys was statistically significant, χ^2^(6) = 17.48, *p* = 0.008, Cramer’s *V* = 0.21, 95% CI for Cramer’s *V* [0.14, 0.31], κ = 0.02, *p* = 0.481, 95% CI for κ [0.01, 0.04]. This relationship was statistically significant and weak, and the agreement between the two types of evaluations of body weight was also weak, due only to the consistency found in the underweight group. For details, see Figure 3a. The relationship between the measured BMI category and the accuracy of weight estimation in girls was statistically significant, χ^2^(6) = 68.10, *p* < 0.001, Cramer’s *V* = 0.46, 95% CI for Cramer’s *V* [0.33, 0.61], κ = −0.05, *p* = 0.006, 95% CI for κ [−0.11, 0.01]; however, the results indicate a weak disagreement between these two types of estimations. For details, see Figure 3b.

There were no significant differences in the reasons for the parents’ misperceptions, except for the child’s actual weight category: normal weight vs. overweight, and normal weight vs. obese. Specifically, the logistic regression analyses summarized in Table 2a,b and Table 3 indicate that neither children’s age, sex, existence of siblings, mother’s and father’s education, nor family affluence were statistically significant predictors of parents’ accuracy in the identifying children’s weight category. Moreover, the parents of both boys and girls were more likely to misperceive their children’s weight if they were obese, and for girls, also if they were overweight (see Table 2b for details). 

## 4. Discussion

The purpose of this study was to investigate the prevalence of childhood obesity and parents’ perception of their children’s weight category, as well as the factors related to the accuracy of parental perception. Here, we provide further evidence of the high prevalence of overweight (OW) and obesity (OB) among children and adolescents (OW: 18.6% and OB: 7.6%), as well as the poor overall parental perception of children’s weight in Poland. Our results suggest that Polish parents cannot properly assess their children’s BMI. Almost one in three parents were unable to correctly identify their children’s weight, with 25.0% underestimating it and 6.0% overestimating it. Results similar to the SOPKARD-Junior study were observed in the Canadian study [28], where 38.0% of parents were unable to correctly interpret their children’s weight level, and in the Portuguese study [29], where 32.9% incorrectly estimated their children’s weight (30.6% underestimated and 2.3% overestimated).

The current results indicate that parents mostly fail to recognize obesity and overweight in their children. Underestimating a child’s weight category may lead to failure in the mitigation of the risk factors associated with weight gain, such as lack of encouragement to perform physical activity and poor monitoring of food intake [30,31,32,33]. Parental misperception of children’s weight has been reported in several studies worldwide; however, it is important to consider that in order to classify BMI, different reference standards have been used, e.g., the National Center for Health Statistics and the Centers for Disease Control and Prevention (CDC), the WHO standard deviations, the International Obesity Task Force (IOTF) standard, and the national BMI percentile grids. The use of different cut-off scores may result in different BMI classifications.

The reasons why parents interpret their children’s weight correctly are not clear; therefore, in our study, we tried to find predictors of the accurate assessment of children’s weight. In some studies, the accurate assessment of children’s weight is related to the child’s BMI, age or gender, as well as parental education and socioeconomic status [34,35]. In our study, the children’s age, sex, existence siblings, mother’s and father’s education, or family affluence did not significantly affect the parents’ perceptions of their children. The only relevant factor related to the assessment was the children’s BMI—if a child was overweight or obese, the chance of a mistake was much higher. Our results are consistent with other reports. In studies conducted in England and the Netherlands, parents have difficulty correctly estimating their child’s weight, regardless of socio-demographic background or the parent’s level of education. This study found that the classification of a child’s weight, as may be expected, is based on the child’s BMI [36,37,38].

A graded effect commonly occurs, i.e., the parents of overweight children consider them to be within the age norm and the parents of obese children consider them to be overweight. In the SOPKARD-Junior study, we observed that parents whose children’s weight category was within the age norm most often defined it accurately *(77*.2%), and more often in girls (83.0%) than in boys (72.5%). The likelihood of the misperception of the weight category increases with the child’s weight, with 49.3% of parents correctly estimating the weight category in overweight children and 42.9% in obese children. In light of the ever-growing problem of overweight and obesity in Poland, the fact that less than half of parents can recognize the problem in their children is highly disturbing.

Our findings, however, cannot demonstrate whether parents are aware of their children’s weight category; nonetheless, they intentionally or unintentionally underestimated it. One of the hypotheses explaining our findings is that cultural factors are involved—social expectations regarding body weight may play a role in the correct assessment of a child’s weight [39]. In some communities, thinness is associated with beauty, while in others, a plump child is considered healthier [40,41,42]. In a cross-sectional multi-center study in eight European countries, parents’ perceptions of their children’s weight varied geographically. Parents from Southern Europe were more likely to misclassify overweight children as “normal weight” (77% in Spain, 70% in Cyprus, and 70% in Italy) compared with parents from Central and Northern Europe (58% in Sweden, and 51% in Hungary) [43].

It also seems that parents may be aware of their children’s excessive weight, but they do not want to stigmatize or label them [43,44]. Such beliefs even have scientific substantiation—the Longitudinal Study of Girls Aged 10 to 19 Years found that individuals who were labeled as “too fat” in childhood were associated with higher odds of having an obese BMI nearly a decade later, regardless of initial BMI [45,46]. In the SOPKARD-Junior study, the fact that the majority of parents were able to correctly identify the BMI of normal children may support the hypothesis that parents are aware of excessive weight, but fear stigmatization or labeling.

Another hypothesis explaining the phenomenon of underestimating children’s weight is that admitting their child is “too overweight” may cause a sense of guilt and failure [43,47]. Recognition of the weight problem would require the parents to make changes to their own lifestyles, which may be another reason for the child’s abnormal BMI not being reported.

It is also possible that parents believe that overweight and obesity is a temporary problem that children will grow out of. Research suggests that parents may not be able to recognize obesity in their child because obesity in children and adults has been standardized [48]. It has been observed that, with the current high prevalence of overweight, the weight norm is now a larger body figure. This means that a parent may underestimate their child’s weight if it is not significantly different from other children in the family or age group. It is noted that the increased prevalence of obesity in the population will lead to an increase in the range of body sizes at which the body is perceived as “normal” [49]. In a study conducted in Italy, one of the factors associated with an underestimation of child weight was if the child lived in a region with high obesity [50]. In another study from Kuwait, which is considered one of the most obese countries in the world according to the WHO ranking, it was found that 77.9% of mothers of overweight children considered their child to be within the age norm and 14.3% considered their child to be underweight. In addition, 45.5% of mothers classified obese children as having a normal body weight [51]. Longitudinal studies have documented that parents’ underestimation of their children’s weight increased concurrently with an increase in the prevalence of obesity in the population [52].

In the SOPKARD-Junior study, almost 50% of parents misclassified girls who were underweight, but none of the boys with underweight were wrongly classified. Cultural factors may play a role in this aspect, as slimness in women is considered an ideal in certain parts of the population [53,54].

The major strength of the current study is the use of objective measures of children’s weight and height, complying with the current measurement standards [29]. The second advantage of the study is the inclusion of data collected in a large panel study, SOPKARD-Junior, conducted in one major region of Poland. This project started in 2006, and every year, a large proportion of the invited parents and children agree to participate [20]; the attendance rate was approximately 90% for several years. Importantly, the current study utilized data from three independent sampling waves: 2017, 2018, and 2019. Consequently, the current study complements the small number of studies on the parental perception of children’s weight in the Polish population. The third strength is the application of a psychometrically validated instrument to measure Family Affluence Status (wealth); as previous studies have shown [26,27], FAS is considered an adequate measure of family affluence in Central European (i.e., post-Soviet) countries.

The major limitation of the study is the simplicity of the instrument used to measure the parental perception of children’s weight. Instead of a silhouette rating scale [40], a questionnaire with five rating options was employed. As the measurement of the parental perception of children’s weight was part of the SOPKARD-Junior panel, it was decided to apply a simple but valid questionnaire. As indicated in other studies [29], this measure fulfills its function. Another limitation is the small sample size in some subgroup analyses, which could have resulted in non-significant results.

## 5. Conclusions

This study contributes to the knowledge concerning the high prevalence of overweight and obesity among children and adolescents in Poland and their parents’ perception of their weight status. The analyses of parental perception indicated that most parents underestimate the weight of overweight or obese children, although underestimation was more common in girls than in boys. Our results suggest that this pattern occurs regardless of the children’s age and sex, the existence of siblings, family affluence, and mother’s and father’s education. This misperception may be a risk factor for the development of obesity.

Parents who misjudge their children’s weight may ignore messages related to the dangerous consequences of a too-high body weight. Therefore, before recommending lifestyle changes, raising awareness in parents should be considered first. Health care professionals should establish earlier contact with the parents of children at risk of obesity. In these children, weight and height measurements should be taken regularly, rather than allowing weight category to be dependent upon parental weight assessments. These suggestions have been adopted as the general strategy in various nations [4,55]. BMI screening programs conducted in the U.S. indicated that the parents of overweight children who received a BMI Report Card were more likely to plan medical care, physical activity, and dietary intervention than parents who did not receive such care. Most parents also believed that an annual screening program should be implemented [56]. Another study found that parents welcome efforts toward physician-facilitated goal setting [57].

## Figures and Tables

**Table 1:** Characteristics of study subjects by sex (*N* = 381 ^1^).

		All		Boys (*n* = 211)		Girls (*n* = 170)		
Demographic Profile		*N*	*%*	*N*	%	*N*	%	d
Children’s sex	Boys	211	55.4	–	–	–	–	
	Girls	170	44.6					
Children’s age	9–10	79	20.7	38	18.0	41	24.1	
	11–12	194	50.9	113	53.6	81	47.7	
	13–14	98	25.7	52	24.6	46	27.0	
	15–16	10	2.7	8	3.8	2	1.2	
	*M*/*SD*	11.67/1.52		11.75/1.56		11.59/1.47		*t*(379) = −1.02, *p* < 0.306
Children’s BMI	Underweight	20	5.2	6	2.8	14	8.2	
	Normal weight	261	68.5	145	68.7	116	68.2	
	Overweight	71	18.6	37	17.5	34	20.0	
	Obese	29	7.6	23	10.9	6	3.5	
								χ^2^(3) = 12.24, *p* = 0.007
Children’s siblings	No	277	79.1	36	18.4	37	24.0	
	Yes	73	20.9	160	81.6	117	76.0	
								χ^2^(1) = 1.67, *p* = 0.196
Mother’s education level	Lower than high school	33	8.7	20	9.5	13	7.6	
	High school	112	29.4	58	27.5	54	31.8	
	University level	236	61.9	133	63.0	103	60.6	
								χ^2^(2) = 1.04, *p* = 0.594
Father’s education level	Lower than high school	52	13.6	26	12.3	26	15.3	
	High school	157	33.3	73	34.6	54	31.8	
	University level	202	53.0	112	53.1	90	52.9	
								χ^2^(2) = 0.36, *p* = 0.658
Family Affluence Scale (FAS)	Lower family affluence	95	24.9	50	23.7	45	26.5	
	Medium family affluence	179	47.0	91	43.1	88	51.8	
	Higher family affluence	107	28.1	70	33.2	37	21.8	
								χ^2^(2) = 6.15, *p* = 0.046

^1^ *N* does not always equal 381, owing to minor missing data. The percentage accounts for valid responses; d = tested differences between boys and girls at the given demographic variable.

**Table 2a:** Predictors of parents’ accuracy in identifying children’s weight category (*n* = 343 ^1^).

Factors		*B*	*SE*	Wald	*p*	OR	95% CI
Children’s age	10–16	−0.07	0.08	0.78	0.378	0.93	[0.80, 1.09]
Children’s sex	Boys (=1)	–	–	–	–	–	reference
	Girls (=0)	0.01	0.26	0.01	0.963	1.01	[0.61, 1.67]
Children’s BMI	Underweight	−0.44	0.53	0.70	0.403	0.64	[0.23, 1.81]
	Normal weight	–	–	–	–	–	reference
	Overweight	−1.20	0.31	15.10	<0.001	0.30	[0.16, 0.55]
	Obese	−1.63	0.45	13.38	<0.001	0.20	[0.08, 0.47]
Children’s siblings	No	–	–	–	–	–	reference
	Yes	−0.14	0.30	0.22	0.641	0.87	[0.48, 1.75]
Mother’s education level	Lower than high school	–	–	–	–	–	reference
	High school	0.53	0.50	1.13	0.287	1.70	[0.64, 4.54]
	University level	0.41	0.53	0.60	0.440	1.51	[0.53, 4.24]
Father’s education level	Lower than high school	–	–	–	–	–	reference
	High school	−0.03	0.45	0.01	0.944	0.97	[0.40, 2.34]
	University level	−0.24	0.48	0.25	0.620	0.79	[0.31, 2.01]
Family Affluence Scale (FAS)	Low family affluence	–	–	–	–	–	reference
	Medium family affluence	0.11	0.32	0.12	0.725	1.12	[0.60, 2.08]
	Higher family affluence	−0.33	0.36	0.86	0.355	0.72	[0.35, 1.45]
	Cox–Snell *R*^2^ = 0.08						
	Nagelkerke *R*^2^ = 0.12						
	χ^2^(12) = 30.24, *p* = 0.003						

^1^ The number of valid responses for the model. Logistic regression (enter method). The dependent variable was the accuracy of the parents’ perception of children’s weight categories (0 = incorrect, 1 = correct).

**Table 2b:** Predictors of parents’ accuracy in identifying children’s weight category, by sex.

		Boys (*n* = 191)			Girls (*n* = 152)		
Factors		OR	95% CI	*p*	OR	95% CI	*p*
Children’s age	10–16	0.97	[0.79, 1.20]	0.781	0.93	[0.71, 1.21]	0.593
Children’s BMI	Underweight	– ^1^	–	–	0.26	[0.08, 0.91]	0.035
	Normal weight	–	reference	–	–	reference	–
	Overweight	0.50	[0.22, 1.15]	0.105	0.16	[0.06, 0.41]	<0.001
	Obese	0.29	[0.11, 0.80]	0.016	0.04	[0.01, 0.41]	0.007
Children’s siblings	No	–	reference	–	–	reference	–
	Yes	2.00	[0.89, 4.51]	0.093	0.62	[0.23, 1.64]	0.332
Mother’s education level	Lower than high school	–	reference	–	–	reference	–
	High school	1.15	[0.29, 4.56]	0.841	2.69	[0.58, 12.47]	0.205
	University level	1.34	[0.32, 5.51]	0.688	1.52	[0.30, 7.86]	0.616
Father’s education level	Lower than high school	–	reference	–	–	reference	–
	High school	0.60	[0.17, 2.14]	0.431	1.74	[0.44, 6.83]	0.431
	University level	0.51	[0.13, 1.96]	0.328	1.30	[0.33, 5.19]	0.710
Family Affluence Scale (FAS)	Low family affluence	–	reference	–	–	reference	–
	Medium family affluence	1.06	[0.44, 2.58]	0.376	1.35	[0.52, 3.47]	0.540
	Higher family affluence	0.65	[0.25, 1.69]	0.191	1.25	[0.37, 4.22]	0.716
	Cox– Snell *R*^2^	0.09			0.18		
	Nagelkerke *R*^2^	0.13			0.25		
	χ^2^(11)/*p*	18.38		0.073	29.51		0.002

^1^ Not calculated correctly due to a lack of cases. Logistic regression (enter method). The dependent variable was the accuracy of the parents’ perception of children’s weight categories (0 = incorrect, 1 = correct).

**Table 3:** Predictors of parents’ accuracy in identifying children’s weight category, by measured BMI.

		Normal Weight (*n* = 235)			Overweight-Obese (*n* = 89)		
Factors		OR	95% CI	*p*	OR	95% CI	*p*
Children’s age	10–16	0.90	[0.74, 1.10]	0.318	1.08	[0.79, 1.48]	0.615
Children’s sex	Boys	–	reference	–	–	reference	–
	Girls	1.58	[0.82, 3.06]	0.171	0.60	[0.24, 1.50]	0.269
Children’s siblings	No	–	reference	–	–	reference	–
	Yes	1.35	[0.61, 2.99]	0.462	1.04	[0.38, 2.85]	0.944
Mother’s education level	Lower than high school	–	reference	–	–	reference	–
	High school	2.48	[0.69, 8.90]	0.164	1.74	[0.33, 9.18]	0.513
	University level	2.22	[0.61, 8.14]	0.230	1.40	[0.21, 9.11]	0.727
Father’s education level	Lower than high school	–	reference	–	–	reference	–
	High school	1.14	[0.35, 3.75]	0.828	0.48	[0.10, 2.25]	0.347
	University level	1.17	[0.32, 4.28]	0.809	0.35	[0.08, 1.56]	0.166
Family Affluence Scale (FAS)	Low family affluence	–	reference	–	–	reference	–
	Medium family affluence	1.11	[0.49, 2.55]	0.800	0.94	[0.29, 3.09]	0.917
	Higher family affluence	0.47	[0.19, 1.12]	0.088	0.43	[0.44, 7.12]	0.427
	Cox–Snell *R*^2^	0.05			0.17		
	Nagelkerke *R*^2^	0.12			0.24		
	χ^2^(9)/*p*	12.79		0.172	5.51		0.809

## Data Availability

The data presented in this study are available on request from the corresponding author. The data are not publicly available due to privacy restrictions.
